# Evaluating species at risk in data‐limited fisheries: A productivity–susceptibility analysis for marine aquarium fish

**DOI:** 10.1002/eap.70272

**Published:** 2026-06-09

**Authors:** Gabrielle A. Baillargeon, Alice A. Wynn, Jemelyn Grace P. Baldisimo, Michael F. Tlusty, Andrew L. Rhyne

**Affiliations:** ^1^ Department of Biological Sciences University of Leeds Leeds UK; ^2^ School for the Environment University of Massachusetts Boston Boston Massachusetts USA; ^3^ Biological Sciences Department Old Dominion University Norfolk Virginia USA; ^4^ Global Ocean Conservation Program Monterey Bay Aquarium Monterey California USA; ^5^ Department of Biology, Marine Biology, and Environmental Science Roger Williams University Bristol Rhode Island USA

**Keywords:** coral reefs, data‐limited fishery, marine aquarium trade, ornamental fish, risk assessment, vulnerability

## Abstract

The marine aquarium trade (MAT) is a significant global industry harvesting millions of wild‐caught, live coral reef fishes for public and private aquaria markets in the United States and Europe annually, while supporting fisher livelihoods in the Indo‐Pacific. This diverse and species‐rich trade is considered data‐limited, creating barriers to quantifying the current and future socio‐ecological sustainability of the fishery. We present a revised and expanded productivity–susceptibility analysis (PSA) that serves as a holistic risk assessment to estimate the vulnerability of marine aquarium fish to overfishing. Our global analysis includes 306 species that are actively in trade. Improvements to the PSA framework from previous research including novel susceptibility factors, methods to overcome missing data for individual species factors, and assessing a large, diverse group of marine fish under a single, targeted assessment framework. Our results show that an overwhelming 81.4% of species evaluated fall into the least or moderately vulnerable classification, while the remaining species (*n* = 57) have higher vulnerability scores designating them as high priority for localized assessment and management initiatives. Most teleost fish in the trade are considered sustainable, while eels and elasmobranchs have the highest vulnerability scores. A comparative case study between our PSA and the popular FishBase vulnerability tool illustrates how the latter can be ill‐suited to handle the data limitations common to nonfood fishes. Our study demonstrates how the PSA is a robust, data‐limited fishery assessment to prioritize species in the MAT for further assessment, monitoring, and management.

## INTRODUCTION

Appropriate fisheries management needs to address the productive capacity of each stock, as well as its susceptibility to overfishing (Hobday et al., 2007). Without the productivity–susceptibility analysis (PSA), adequate management actions may be challenging to implement. Fisheries that target marine aquarium trade (MAT) species are recognized for their diversity and often small harvest volumes (Rhyne et al., 2017). Due to the high diversity and low volume characteristic of this fishery, most species in the MAT remain wild‐caught (Tlusty et al., 2013). Although there have been recent technological advancements paving the way for aquaculture to supplement wild‐caught fish, there remains a limited number of species where commercial aquaculture is viable (Tlusty, 2002). Commercial aquaculture is concentrated in western countries that have historically played the role of major importing nations in the trade, instead of supplementing fishing livelihoods with cultured production in native ranges (Tlusty, 2002).

Rhyne et al. (2012, 2017) reported that the United States is the largest importer of marine aquarium fish, having imported approximately 2300 species from over 40 countries within a 4‐year period. Similar trends in imports are observed in the UK and the European Union (Rhyne et al., 2017). However, it is notable that only a limited number of these species have undergone assessment or are covered by active management plans to evaluate their vulnerability to overfishing. Data limitations often arise given the significant effort required to monitor a large number of species being harvested, along with the multitude of biological, environmental, and anthropogenic factors that can influence stock sizes (Baillargeon et al., 2020; Hobday et al., 2007; McClanahan et al., 2023).

Species harvested for the MAT are particularly data‐deficient as they are: (1) less voluminous in trade compared to food fish, (2) often harvested from remote, biodiversity hotspots throughout the coral triangle, (3) catch reporting is severely limited at most points in the supply chain leading to a near complete lack of long‐term datasets within exporting nations (Baillargeon et al., 2020; Biondo & Burki, 2020; Dee et al., 2014, 2019; Fujita et al., 2014; Okemwa et al., 2016; Wood, 2001a, 2001b). Most fish for the MAT are caught within the Indo‐Pacific (Rhyne et al., 2017), often in remote island locations resulting in multiple points of sale prior to reaching the final exporting port. Key exporting nations such as the Philippines and Indonesia are characterized by decentralized government structures that lack the resources to effectively enforce fishery management regulations that do exist, such as gear restriction of a universal ban on cyanide fishing. Data limitations are exacerbated by a lack of uniform reporting required at any point in the supply chain, rendering scientists unable to easily trace fishery export volumes back to the source and discern if the level of harvest poses a risk to reef fish populations present.

A successful method to rapidly assess the vulnerability of a species due to fishing activity in a data‐limited context is the PSA (Hobday et al., 2007, Patrick et al., 2009). This data‐limited fisheries assessment is widely utilized by food fisheries when sufficient information on catch, effort, gear type, and species biomass data is unavailable thus prohibiting a stock assessment. The PSA estimates a stock's productivity based on widely known life history traits that are tailored to key growth indicators and trophic niches specific to a group of fishes. To assess fishery and trade influences on a stock's abundance, the PSA also scores the susceptibility of a fish to fishing pressure across several factors. A single vulnerability score is generated based on the combination of productivity and susceptibility scores (Hobday et al., 2007, 2011). The MAT PSA developed by Baillargeon et al. (2020) focused on 32 species, comprising the top 20 traded species along with 12 species assessed by other PSA studies. Seven productivity and five susceptibility factors were deemed robust across all species and were used to calculate vulnerability (Baillargeon et al., 2020).

In 2019, parties of the Convention on International Trade in Endangered Species of Wild Fauna and Flora (CITES) agreed to conduct a review and technical workshop to help understand trends in the MAT and identify species at risk of overexploitation, highlighting the need for a robust and accessible assessment method for managers to implement at the local to global scale (CoP19 Inf. 99). At the 2024 CITES technical workshop for marine ornamental fish species in Geneva, the outcomes emphasize the need to invest in research into the best analytical method for species prioritization (AC33, Document 44). Throughout this process, the ease and accessibility of FishBase's (Froese & Pauly, 2023) vulnerability calculation was highlighted as a leading tool to evaluate the MAT (UNEP‐WCMC, 2022). However, FishBase's current model has limitations as a resource for fishery vulnerability information across species, where data may be missing (Cheung et al., 2005). FishBase models are tuned toward species important for food systems, and vulnerability scores have been shown to be ineffective at modeling risk for reef fishes on a global scale and when assessing species which do not conform to growth patterns the model is based on (Go et al., 2015). In response, there has been greater consideration of the PSA as an alternate, more accurate model to evaluate the risk fishing poses to species in the MAT.

To ensure appropriate resource extraction of the MAT species and sustainability of the MAT, it is critical to perform rapid fisheries assessments that provide accurate results and do not require extensive fisheries management resources. Therefore, we expanded the PSA model developed by Baillargeon et al. (2020) to 306 species of diverse taxonomic backgrounds and that represent the majority of all commonly traded fish. We addressed the issue of data deficiency by implementing methods to overcome data gaps, quantified factors that are most impactful to a fish's vulnerability across the diverse 306 species, and improved the specificity of the PSA framework and vulnerability characterization to marine aquarium fish.

## MATERIALS AND METHODS

### Species selection

**FIGURE 1:**
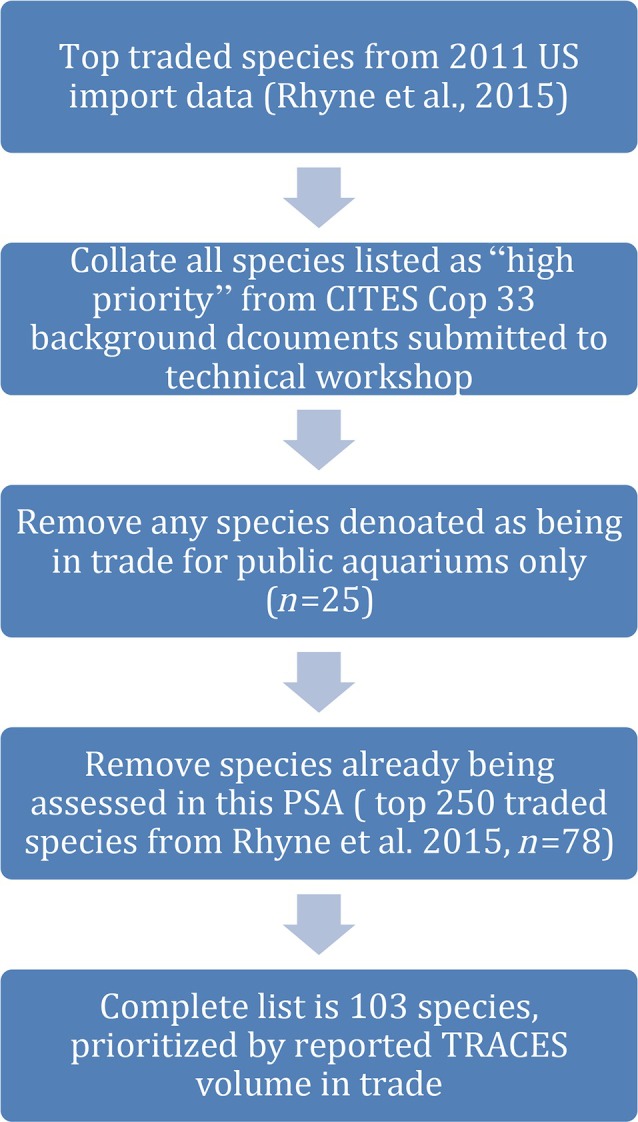
Decision flow chart of species to include and exclude in this productivity–susceptibility analysis based on available data sources (Rhyne et al., 2015, and CITES AC33 Document 44).

A large subset of the species represents the top traded species into the United States from the most recent available scanned invoices (2011). This resulted in identifying the top 306 species in trade in volume, an estimated 92.5% of all species imported into the United States (Rhyne et al., 2015). Additional species (*n* = 48) were added to the list based on several background documents that were submitted at the 2023 CITES technical workshop which used different methods to identify high priority species for parties to consider (CITES AC 33 Doc 44, Rev. 2). By collating high priority species from all submitted documents, a list of approximately 200 fish was produced. After removing species already included in the top 306 most traded list (*n* = 75), along with those that were noted to be exclusively traded for public aquariums (*n* = 25), the final list consisted of 103 species to be included in the global PSA (Figure 1). Species were then further prioritized for inclusion based on their documented number of individuals recorded in the TRACES database. In total, 306 species were evaluated using this PSA framework.

### Assessing variables to include in PSA

Identifying the optimal set of factors to obtain the vulnerability score and mitigate uncertainties determines the effectiveness of a PSA framework. To achieve this, the PSA model developed by Baillargeon et al. (2020) was applied to 306 species documented to be actively in the MAT (Rhyne et al., 2015, 2017). The feasibility and practicality of use were evaluated by assessing data availability for each productivity and susceptibility factor. When species‐level data were unavailable, a congener, closest genetic relative, or family level data were used. In the absence of data, experts on reproductive biology of marine ornamental fish species were consulted to provide fecundity data needed for this study.

Productivity and susceptibility factors were scored for each species individually on a 1–3 (low–high) scale, in line with Patrick et al. (2009) and Hobday et al. (2011) (see Appendix S1: Table S1 for scoring matrix). Productivity is an indirect measurement of a species’ ability to reproduce and indicates resiliency to changing environmental conditions (Baillargeon et al., 2020). Five productivity factors: maximum size, mean trophic level, breeding strategy, fecundity, and pelagic larval duration (PLD) were included in this PSA framework (Table 1).

**TABLE 1:** Productivity and susceptibility factors with corresponding data sources.

Factor	Data sources
Productivity	
**Maximum size**	Primary literature, Michael (2005), FishBase
Mean trophic level	FishBase, primary literature
Breeding strategy	FishBase, Breder and Rosen (1966), Thresher (1984)
**Fecundity**	Primary literature, hobbyist forums (i.e., breedersregistry.org, mbisite.org), FishBase
Pelagic larval duration	Primary literature
Susceptibility	
**Ecological niche/distribution**	Eschmeyer's catalog of fishes, IUCN Red List Assessments, FishBase
Cyanide use	Aquariumtradedata.org
Encounterability depth	IUCN Red List Assessments
Aquarium suitability	Online hobbyist databases, (LiveAquaria.com, Saltcorner.com, Reeflex.net), Michael (2005)
**Trade volume**	Aquariumtradedata.org
**Life cycle stage of harvest**	LiveAquaria.com, BlueZooAquatics.com

*Note*: Bold text indicates a factor weight of 2 in the model.

Susceptibility measures the likelihood that fishing pressures will have a negative impact on a species’ population (Patrick et al., 2009). Susceptibility has a reverse scale from productivity, where high susceptibility translates to a higher vulnerability score. This artifact was introduced by Patrick et al. (2009), and we continue this use so PSA scores are available for comparison in a meta‐analysis. Six susceptibility factors: geographic distribution, encounterability depth, suitability for aquarium keeping, volume in trade, life stages harvested, and cyanide were included in this PSA framework (Table 1).

Model sensitivity was also tested by looking at the influence of each productivity and susceptibility factor on the resulting vulnerability score. Validation of the method developed by Baillargeon et al. (2020) resulted in changes to the productivity and susceptibility factors being assessed and the weighting for each factor (see Appendix S1: Table S1 for complete data binning and scoring matrix). An analysis of change in vulnerability scores across the 32 species analyzed in Baillargeon et al. (2020) utilizing the present model was also conducted (Appendix S1: Figure S1). Additionally, a full PSA was run with and without volume in trade as a susceptibility factor, to understand the impacts of including trade data in this type of assessment.

Validity of scientific names for species included in this study was checked against the online version of Eschmeyer's Catalog of Fishes up to December 2023 (Fricke et al., 2023). Data for all species were sourced from a mix of primary literature, open‐source databases and repositories, and aquarium hobbyists’ gray literature (Table 1).

A phylogenetic tree with vulnerability score heatmap data was created using the *rtol*, *ape*, and *ggtree* packages in R (Appendix S1: Figure S2; Wynn, 2025 at https://doi.org/10.5281/zenodo.14833664). Organizing the data phylogenetically helps to visualize vulnerability score trends across taxonomic levels. Phylogenetic data were sourced from the Open Tree of Life database.

### PSA mathematical framework

Productivity was calculated using a weighted arithmetic mean based on six life history factors, where *x*_i_ is the factor score and *b*_i_ is the weight (Table 1). Increasing the factor weight to 2 represents the factor's importance in determining the vulnerability of a species in a fishery.

(1) $$p=\frac{\sum_{i=1}^{6}x_{i}\times b_{i}}{\sum_{i=1}^{6}b_{i}}$$

Susceptibility was calculated by using a weighted mean of logarithms expressed as an exponential function, with base 10 raised to the power of the weighted logarithmic mean, where *y*_*i*_ is the susceptibility factor score and *a*_*i*_ is the factor weight:

(2) $$s=10^{\frac{\sum_{i=1}^{5}\log\;y_{i}\times a_{i}}{\sum_{i=1}^{5}a_{i}\;}}$$

These factors were quantified in a data‐binning process where productivity and susceptibility scores are calculated separately then inputted into the Euclidean distance formula to output the vulnerability score (*v*). Here *p* is the mean productivity score, *s* is the mean susceptibility score, and *v* is the vulnerability score (Equation 3). Vulnerability is functionally the distance from the origin (1, 3) of the productivity–susceptibility plot.

(3) $$v=\sqrt{{\left(p-3\right)}^{2}+{\left(s-1\right)}^{2}}$$

### Productivity and susceptibility factor refinement

#### Productivity factors

Length at maturity and maximum age factors were used in Baillargeon et al. (2020) but were removed in this analysis due to lack of reliable data for a majority of the 306 species being assessed. Maximum size was closely correlated to the maximum age and length at maturity (*R* ^2^ > 0.9); to reduce redundancy and improve species‐level data inputs, maximum size remained. Maximum size was given double weight among the productivity factors to account for its representation of numerous factors.

The factor breeding strategy was adjusted to be dependent on fecundity in cases of high disparity between breeding strategy and fecundity values to ensure accuracy (Appendix S1: Table S2). Because of this scaling, score weight for this factor was set to 1 instead of 2 to avoid double‐counting within the model. All other factors and scoring bins remained unchanged from Baillargeon et al. (2020).

#### Susceptibility factors

**FIGURE 2:**
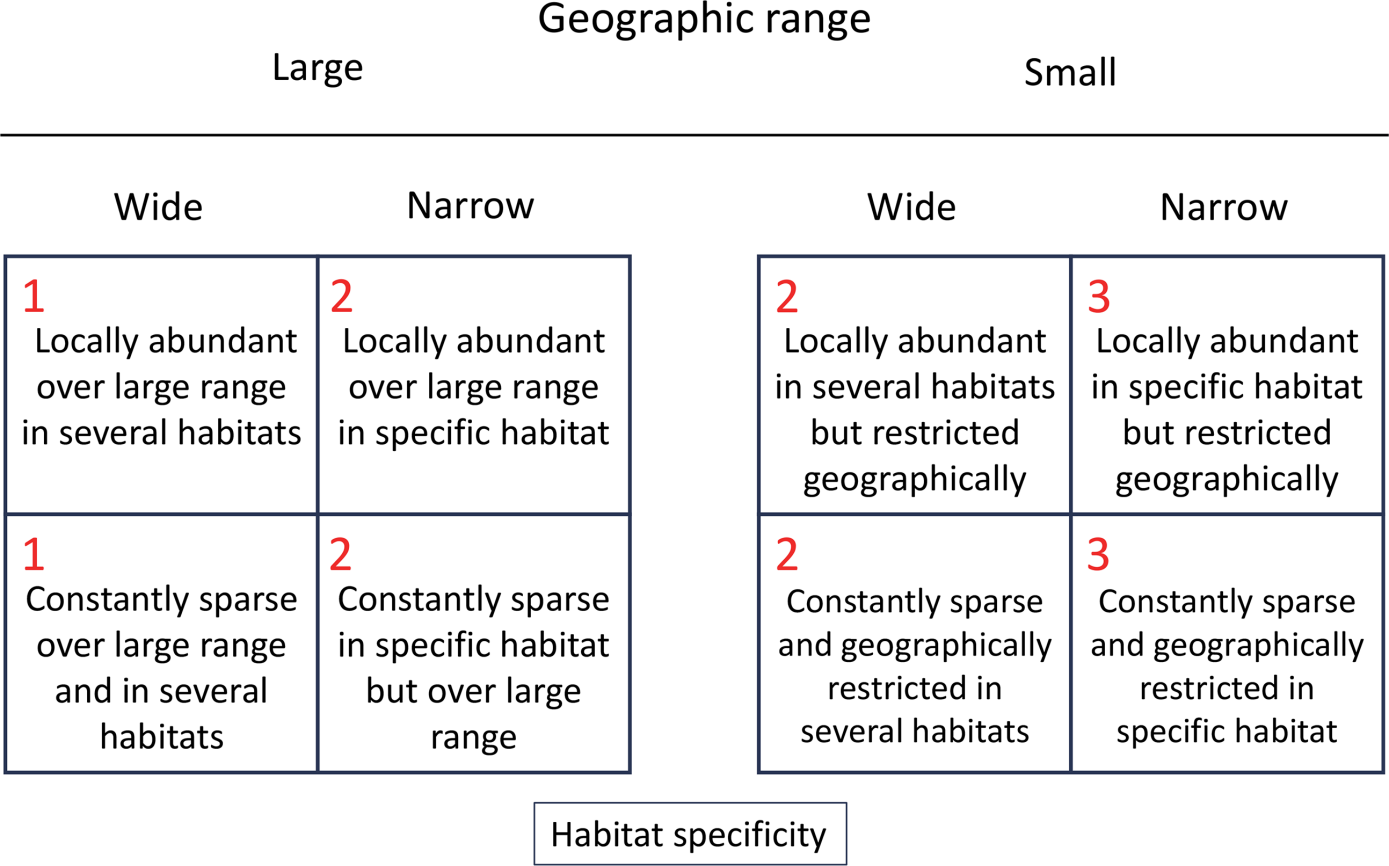
Scoring matrix for susceptibility factor ecological niche/distribution based on Seven Forms of Rarity (Rabinowitz, 1981). Ecological niche/distribution scores were calculated based on their geographic range determined by the California Academy of Science and IUCN databases, combined with habitat specificity from FishBase. Factor score is indicated in the upper left corner of each categorization bin.

Ecological niche and geographic distribution were combined into a single factor in this model, to account for the interaction of species range and habitat specificity impacting overall susceptibility to fishing effort. Geographic distribution (categorized as large or small) was determined by referring to published IUCN Red List Assessments (IUCN, 2023) and Eschmeyer's Catalog of Fishes (Fricke et al., 2023). A small geographic distribution was limited to a single nation or small group of nations, whereas a large distribution would be classified as an entire region to global distribution. This was then cross‐referenced with habitat specificity information from FishBase (Froese & Pauly, 2023), which was categorized as wide or narrow. Data from ecological niche and geographic distribution were then binned into a single‐factor score following Rabinowitz (1981) methodology (Figure 2, Appendix S1: Table S1). For example, species with small geographic ranges and narrow habitat specificity were classified as most susceptible, with a score of 3.

Encounterability depth was obtained from the depth range of each species indicated in the IUCN Red List database (IUCN, 2023) and scored based on the maximum depth instead of the average depth (Baillargeon et al., 2020) where a species can be observed.

Aquarium suitability was scored by converting descriptive care levels estimated by hobbyist sources (Table 1) into a 5‐point numerical scale (least difficult = 1 to most difficult = 5) and scored across three data sources. These hobbyist databases provided various phrases used to describe care difficulty (Appendix S1: Table S1). This 5‐point scale was converted to the 3‐category PSA framework by assigning 1 for average of care scores <2.5 (Less difficult), 2 for scores between 2.5 and 3.5 (Moderate difficulty), and 3 for scores >3.5 (More difficult). This gives a quantifiable range to narrative data, in comparison to the “easy, medium, hard” categorization from the same data sources in Baillargeon et al. (2020).

When fishes are harvested at a rate faster than their reproduction rate, overexploitation occurs (Aubone, 2004). Thus, species with a high volume in trade and high fecundity may be considered less susceptible to overexploitation compared to species with low to moderate volume in trade and low fecundity. To capture this in the revised PSA model, the factor score for trade volume was scaled based on the species’ productivity score. Data bins are based on the first and third quartiles from the spread of 2011 volume in trade data. This factor was weighted at 2 due to the importance of trade volume on fishery vulnerability (Table 1; Appendix S1: Table S1).

The life cycle stage at harvest (LCSH) was added as a susceptibility factor due to its impact on local population dynamics. Harvesting adults negatively impacts the reproductive potential of a stock, including its ability to withstand and recover from fishing pressure (Begg & Marteinsdottir, 2003; Law, 2007). Fishing juveniles may reduce the population of breeding adults in the future and affect population renewal (Wood, 2001a). As such, species harvested in both juvenile and adult stages were considered most susceptible to overfishing (score of 3) compared to species harvested only as juveniles (score of 1) or adults (score of 2). This scoring framework was developed based on the idea that removing all life stages significantly reduces potential recruitment, as does removing adult broodstock, whereas removing nonbreeding juveniles has less direct impact on population size (Appendix S1: Figure S3). The LCSH on a per species basis was confirmed by referencing size classes of fish against size at maturity lengths, as well as if the species was being sold as an adult, juvenile, or both on well‐known online wholesalers (Liveaquaria.com, QualityMarine.com, Bluezooaquatics.com). In the absence of available data, an expert in aquarium fish rearing and trade was consulted. Systematic scoring details for life stage at harvest based on publicly accessible data found on retailer sites is shown in Appendix S1: Figure S3.

### Sensitivity analysis and model comparison

A model sensitivity analysis was done to test the impacts of individual factor scores, weighting, and combined effects across multiple factors for both productivity and susceptibility. Multiple factor scores were manipulated, individually and then in succession, while all other factors were set at a neutral score of 2 (Appendix S1: Figure S4). This analysis compared the change in vulnerability score across three weighted and unweighted factors. This three‐factor manipulation included an equal number of weighted (1) and unweighted factors (2) across productivity and susceptibility factors. An expanded model sensitivity analysis was conducted to reveal trends across single weighted and unweighted factors, with all other factors set at a neutral score of 2 (Appendix S1: Table S3). Single productivity factors manipulated include maximum size and trophic level, while susceptibility factors manipulated include ecological niche + distribution and aquarium suitability.

PSA species outcomes were directly compared with the widely available and open‐access repositories and assessments for such a large volume of species: IUCN Red List and FishBase. The conservation status of the 306 species in this study was retrieved from the IUCN Red List website (IUCN, 2023) and categorically compared to the sustainability categorization of PSA vulnerability scores based on our clustering methods (Baldisimo, 2025). Vulnerability scores were scraped from FishBase.org for all species using R.

### Gaussian mixture modeling to classify species vulnerability

A semi‐supervised machine learning algorithm was implemented to classify all 306 species evaluated into three distinct vulnerability clusters: “most vulnerable,” “moderately vulnerable,” and “least vulnerable.” Multivariate, finite Gaussian mixture models (GMMs) were evaluated for best model fit using the open‐source *R Mclust* package. The silhouette coefficient was evaluated to determine the optimal number of clusters for the expanded dataset. Log‐likelihood and Akaike information criterion (AIC) model comparison led to the selection of the Mclust VVI finite GMM (Appendix S1: Table S4 and Figure S5). The main distinction between this GMM and the former GMM (Baillargeon et al., 2020) is clustering based on productivity, susceptibility, and vulnerability scores instead of only along the *x*–*y* axes. Further, this GMM framework is set to cluster diagonally from the origin while allowing the volume and shape of each cluster ellipse to differ instead of holding size and shape. Full model code is available in gbaillargeon (2026) at https://doi.org/10.5281/zenodo.18941035.

Additionally, a univariate with equal variance GMM was applied to the FishBase vulnerability scores to compare species clustering results between the two frameworks. This type of GMM was chosen using Mclust to select the model that had the lowest negative log‐likelihood.

## RESULTS

**FIGURE 3:**
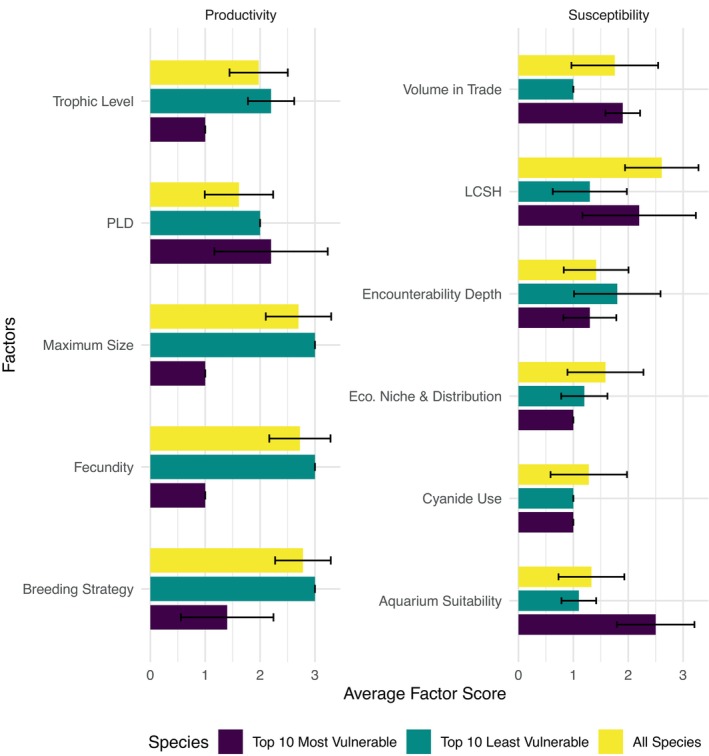
Comparative analysis of average productivity and susceptibility factor scores. Average factor scores (± SD) across five productivity and six susceptibility factors for most vulnerable species (*n* = 10), least vulnerable species (*n* = 10), and all species (*n* = 306).

Across the 306 species, productivity scores ranged from 1.00 to 3.00, and susceptibility scores ranged from 1.00 to 2.32, resulting in a vulnerability range of 0.19–2.03 (Table 2). Significant variation in average factor scores between high and low vulnerability scoring species was observed (ANOVA, *p* < 0.05). For productivity factors, maximum size and fecundity represented the largest difference in average values of fish in the top 10 most and least vulnerable clusters (Figure 3).

**TABLE 2:** Species, family, productivity (*P*), susceptibility (*S*), vulnerability (*V*), rank in trade, IUCN status, availability as captive‐bred for 10 of the most and least vulnerable species assessed.

Species	Family	*P*	*S*	*V*	Rank in trade	IUCN status	Availability as captive‐bred in 2023
Top 10 most vulnerable species							
*Taeniura lymma*	Dasyatidae	1.29	1.13	**1.72**	N/A	LC	Yes
*Aetobatus narinari*	Aetobatidae	1.29	1.17	**1.72**	N/A	EN	No
*Chiloscyllium plagiosum*	Hemiscylliidae	1.29	1.42	**1.77**	N/A	NT	Yes
*Himantura uarnak*	Dasyatidae	1.29	1.49	**1.78**	N/A	EN	No
*Chiloscyllium punctatum*	Hemiscyllidae	1.29	1.61	**1.82**	229	NT	Yes
*Echidna catenata*	Muraenidae	1.29	1.61	**1.82**	N/A	LC	No
*Gymnomuraena zebra*	Muraenidae	1.29	1.61	**1.82**	N/A	LC	No
*Atelomycterus marmoratus*	Atelomycteridae	1.29	1.68	**1.85**	N/A	NT	Yes
*Myrichthys maculosus*	Ophichthidae	1	1.32	**2.03**	N/A	LC	No
*Myrichthys colubrinus*	Ophichthidae	1	1.36	**2.03**	N/A	LC	No
Top 10 least vulnerable species							
*Chrysiptera unimaculata*	Pomacentridae	2.86	1.13	**0.19**	188	LC	No
*Amblypomacentrus breviceps*	Pomacentridae	2.71	1.00	**0.29**	208	LC	No
*Chromis opercularis*	Pomacentridae	2.71	1.00	**0.29**	165	LC	No
*Neoglyphidodon nigroris*	Pomacentridae	2.71	1.08	**0.30**	37	LC	Yes
*Pomacentrus amboinensis*	Pomacentridae	2.86	1.28	**0.31**	125	LC	Yes
*Neoglyphidodon oxyodon*	Pomacentridae	2.71	1.13	**0.31**	49	LC	No
*Chrysiptera rollandi*	Pomacentridae	2.71	1.17	**0.33**	N/A	LC	No
*Dischistodus prosopotaenia*	Pomacentridae	2.71	1.17	**0.33**	237	LC	No
*Amblyglyphidodon curacao*	Pomacentridae	2.71	1.26	**0.39**	86	LC	No
*Amblyglyphidodon ternatensis*	Pomacentridae	2.71	1.26	**0.39**	100	VU	Yes

*Note*: Vulnerability scores are in bold to highlight that is the single final score for that species. Rank in trade of 1 represents the most imported fish into the United States in 2011 (Rhyne et al., 2015), if listed as N/A the species did not appear in the top 306 species from Rhyne et al. (2015). Captive‐bred availability of species is based on Coral Magazine 2023 Captive‐Bred List (CORAL Magazine & Marine Breeding Initiative, 2023) with the categories: Has been successfully bred in captivity, scare, moderate, or common availability. IUCN status in descending order of vulnerability: *Endangered* (EN), *Vulnerable* (VU), *Near Threatened* (NT), and *Least Concern* (LC).

For susceptibility factors, volume in trade and life stage at harvest showed the greatest difference between top 10 most and least vulnerable species (Figure 3). Ecological niche + distribution, cyanide use, and PLD had the least change in average score when comparing the highest and lowest scoring species in terms of vulnerability, indicating these factors had the least influence on the model output (Figure 3). If we assess the most traded species by looking at the top 25% of species in the trade by import quantity, this corresponds to volume in trade rank 1–64 (2011 US import data, Rhyne et al., 2015) where 15,000 individuals or more are traded annually. Within this commonly traded group, 57.8% of species fall into the “least vulnerable” category. Only four “most vulnerable” species are traded at this volume (*Pterapogon kauderni*, *Sphaeramia nematoptera*, *Paracanthurus hepatus*, and *Plectorhinchus chaetodonoides*), accounting for about 7% of the total species considered most vulnerable by our PSA.

**FIGURE 4:**
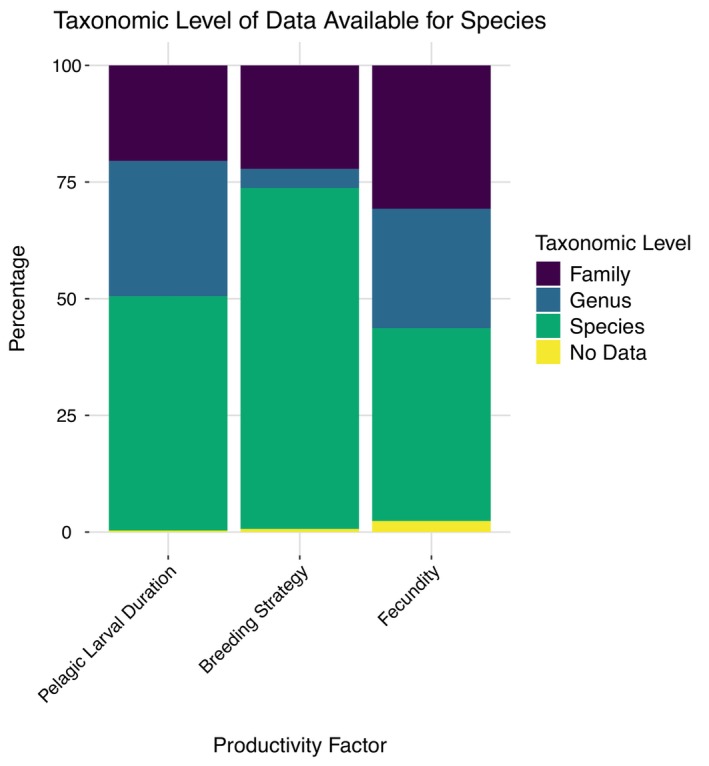
Percentage breakdown of taxonomic level of data available for data‐limited productivity factors (pelagic larval duration, fecundity, and breeding strategy). For all species assessed (*n* = 306), data for factor scoring was drawn from either the species, family, or genus level based on data availability for each species. In rare cases, data were unavailable at any taxonomic level and was denoted as “No Data.” For fecundity, experts on reproductive biology of marine ornamental fish species were consulted when data were missing at the species, genus, or family level.

Except for productivity factors: breeding strategy, PLD, and fecundity, every factor evaluated in this PSA has species‐level data available. For breeding strategy, 72.2% of fish evaluated have species‐level data, where PLD has 50.3%, and 40.2% of fecundity level data (Figure 4). Fecundity has the largest combination of all data types, where breeding strategy was largely determined by species and family level estimates exclusively.

**FIGURE 5:**
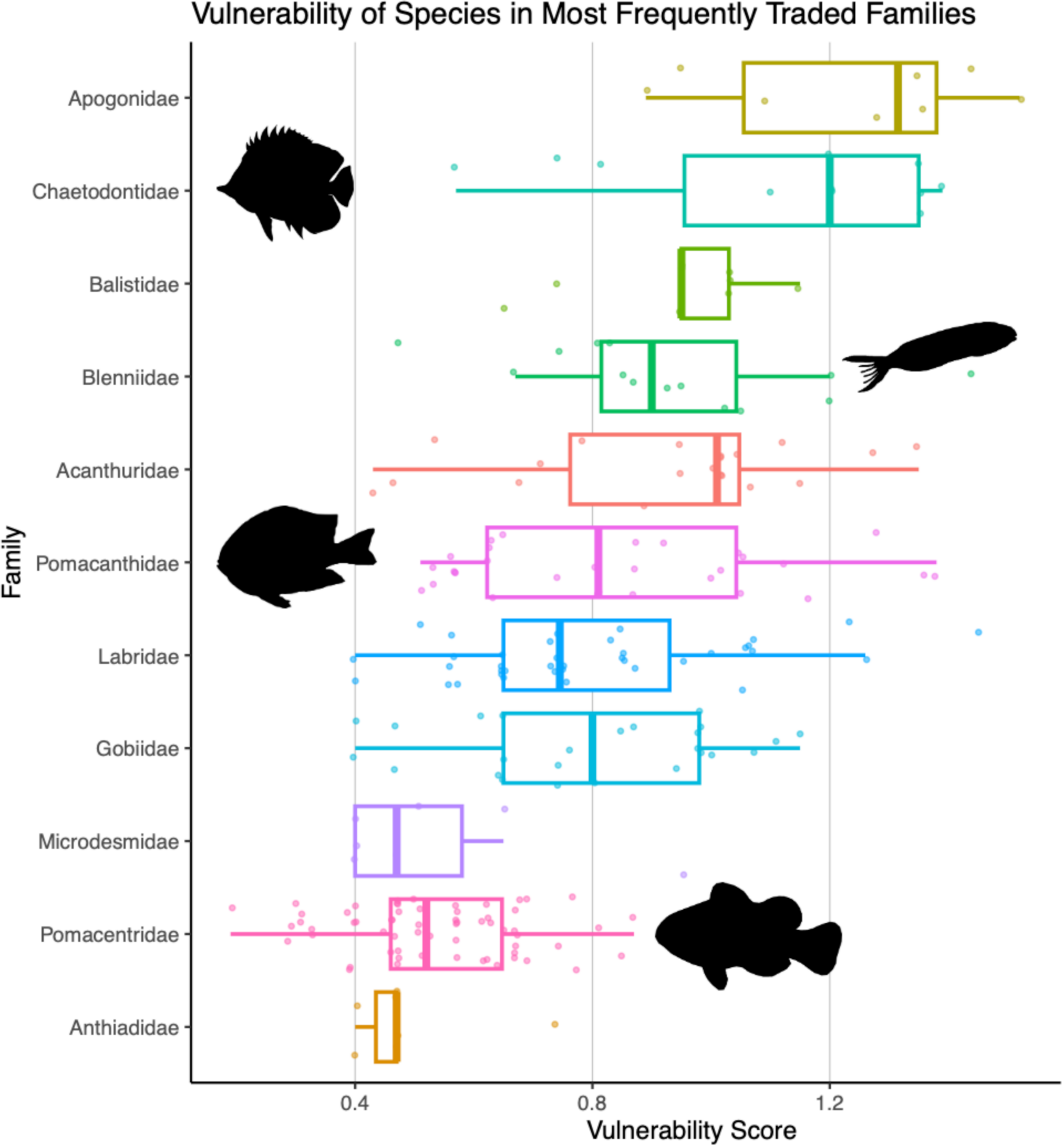
Boxplot of vulnerability scores for the top families by volume in the trade (*n* = 11), ordered from most to least vulnerable. Silhouette icons are of species from the represented families (public domain images sourced from PhyloPic, https://www.phylopic.org/).

A total of 43 families were assessed, and one‐third of those families contained only a single traded species. Notably, seven families (17% of all families evaluated) account for 65% of all species evaluated in this study. The damselfishes (*Pomacentridae*), the largest family of fish in the analysis, have the lowest average vulnerability of the top seven families (*v* = 0.53 ± 0.15, *n* = 62). When examining the top 10 least vulnerable fish (*v* = 0.19–0.39), every species is from family *Pomacentridae*. Whereas the cardinalfishes (*Apogonidae*) are the highest scoring group of fish in terms of vulnerability (*v* = 1.25 ± 0.22, *n* = 18), followed by the butterflyfishes (*Chaetodontidae*) with the second highest average vulnerability scores (*v* = 1.08 ± 0.28, *n* = 10) (Figure 5).

**FIGURE 6:**
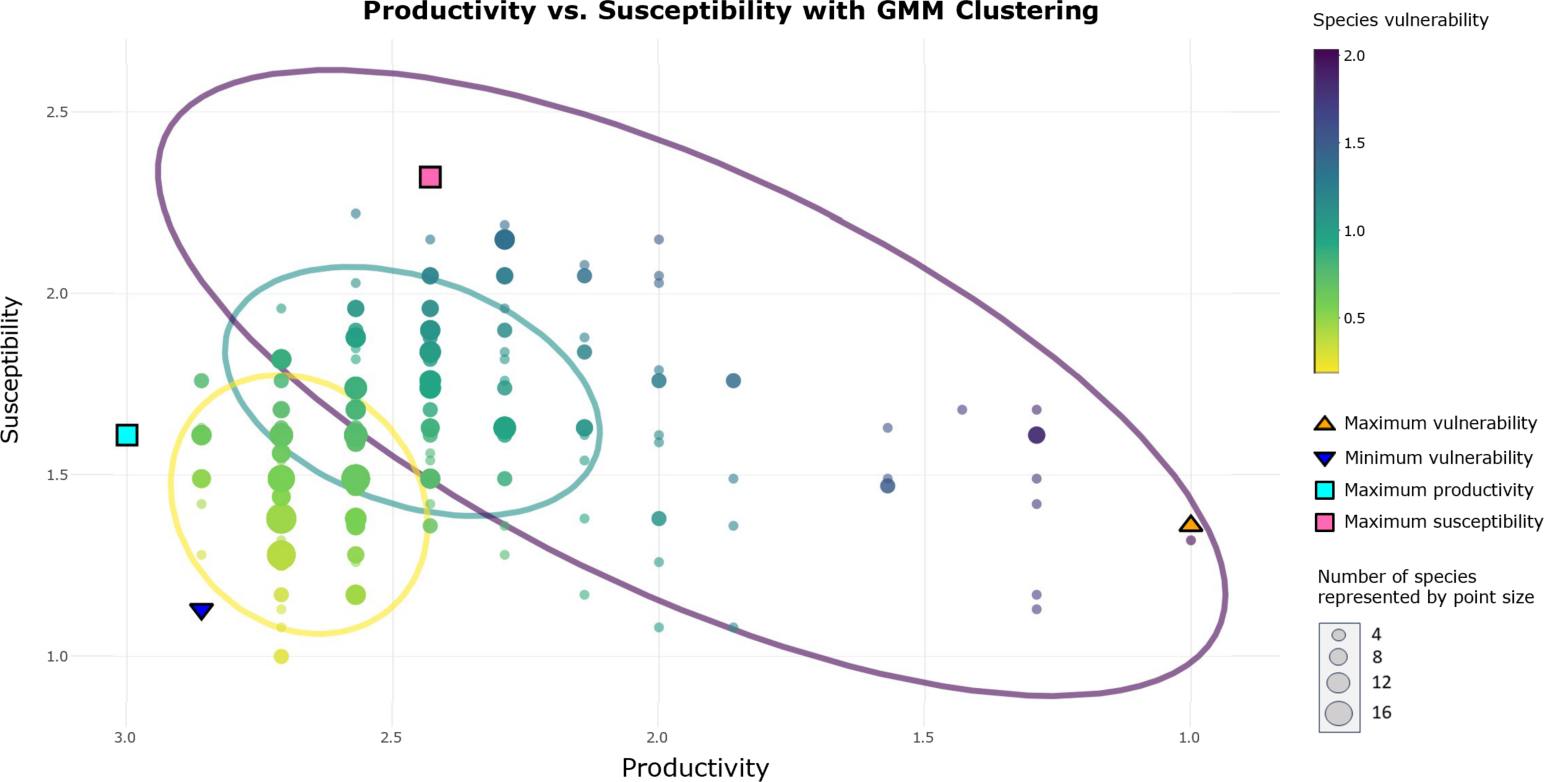
Model sensitivity analysis showing the impact of changing factor scores on the vulnerability score. Species in this analysis are represented by points (*n* = 306) that are colored along a vulnerability gradient. Distinct points represent the minimum and maximum possible vulnerability scores, as well as the discrete maximum productivity and susceptibility scores individually. Icons of fish species of interest (*Amphiprion ocellaris*, *Pterapogon kauderni*, *and Chiloscyllium punctatum*) are displayed on the graph where they fall within the productivity–susceptibility analysis (PSA). Silhouette icons are of species from the represented families (public domain images sourced from PhyloPic, https://www.phylopic.org/).

The model sensitivity analysis highlights that productivity has a greater effect on the vulnerability score when manipulating a group of factors as opposed to susceptibility. All factors aside from those being manipulated were set at a “neutral score” of 2. All scores set to neutral produce a vulnerability score of 1.55 for the example species. Shifting a group of productivity factors (fecundity, breeding strategy, and PLD) from 1 to 3 results in a vulnerability score decrease of 0.88, while a scoring shift of 3 to 1 for a group of susceptibility factors (aquarium suitability, encounterability depth, and LCSH) results in a 0.73 decrease in vulnerability score (Figure 6).

In the expanded sensitivity analysis, manipulation of single factors revealed a marginal tendency for susceptibility to have a greater effect on the vulnerability score opposed to productivity. Shifting a single weighted productivity factor (maximum size) from 1 to 3, and weighted susceptibility factor (ecological niche + distribution) from 3 to 1 resulted in a vulnerability score decrease of 0.36 and 0.39, respectively. Shifting of single unweighted productivity and susceptibility factors (trophic level and aquarium suitability) using the same parameters resulted in a vulnerability score decrease of 0.18 and 0.2, respectively (Figure 6).

The silhouette coefficient (SC = 0.41) determined that three clusters provided the optimal fit for the data when using elliptical shaped clusters that grouped species around their productivity, susceptibility, and vulnerability values. When comparing Mclust GMM models, the log‐likelihood and BIC values indicated that the “VVI” clustering method is the best fit for our data (Appendix S1: Table S4 and Figure S5; BIC = 170, log‐likelihood = 142.24). This resulted in three vulnerability clusters that have significantly different centroids (ANOVA, *p* < 0.01).

**FIGURE 7:**
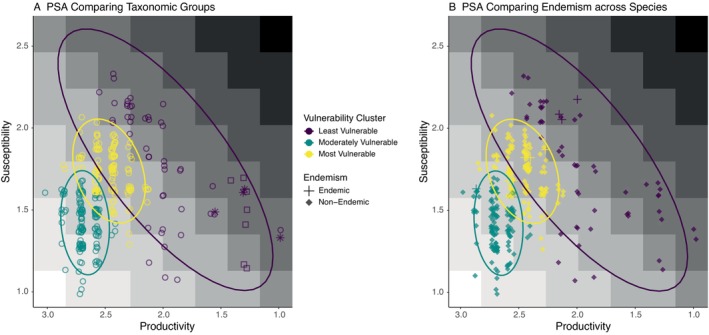
Productivity–susceptibility analysis with Gaussian mixture model clustering designating vulnerability categories for all species assessed (*n* = 306). Distribution of vulnerability scores across taxonomic groups (A) are compared to endemisim across species (B). Circles represent teleots, squares are elasmobrances, and astericks are anguilla taxonomic groups. Color gradient represents vulnerability scores with low to high vulnerability scores corresponding with light to dark gradation diagonally from the origin. The size of data points indicates the number of individuals with that vulnerability score (see legend). Icons in the legend are credited to Microsoft Powerpoint.

Each cluster represents three distinct vulnerability risk groups shaped by the range of productivity and susceptibility scores within that group. The first group is considered least vulnerable due to their characteristic high productivity and low susceptibility scores (HPLS), followed by a moderately vulnerable group (MPMS), and finally, the vulnerable group is defined by low productivity and high susceptibility (LPHS) (Figure 7). The GMM shows the HPLS cluster contains 133 species, the MPMS cluster contains 116 species, and the LPHS cluster contains 57 species (Figure 7). The range of vulnerability scores across the clusters is: 0.19–0.74 for HPLS, 0.74–1.15 for MPMS, and 0.87–1.82 for LPHS, the most vulnerable group. The HPLS and MPMS clusters of the GMM represent fish species that are low priority for further assessment, accounting for 81.3% of species evaluated. The remaining 57 species (18.7%) compose a group of fish that are highest priority for further assessment and management as they could be most vulnerable to fishing of the species assessed here.

The IUCN Red List is widely used as a tool for conservation actions and priorities (IUCN, 2023). A majority of the top 306 MAT species are classified as nonthreatened in the Red List, with 268 species listed as *Least Concern*. However, there are six species categorized as *Near Threatened*, nine as *Vulnerable*, and five *Endangered* (IUCN, 2023). The Red List process uses five criteria to determine a species’ probability of extinction compared to the PSA method that has 11 Productivity and Susceptibility factors specific to assessing vulnerability to overfishing. The PSA for the 306 MAT species included 18 that were considered IUCN *Data Deficient* by the Red List (IUCN, 2023). When IUCN Red List results are compared to the PSA, key differences can be noted including the number of factors used, the PSA's application for data‐limited species, and its ability to measure the threat posed by the MAT on species populations.

All eels and elasmobranchs assessed (*n* = 13) fall within the most vulnerable cluster and account for the top 10 highest vulnerability scores. Within the top 10 most vulnerable species, there are three *Near Threatened* species (*Chiloscyllium plagiosum*, *Chiloscyllium punctatum*, and *Atelomycterus marmoratus*), two *Endangered* species (*Aetobatus narinari*, *Himantura uarnak*), and the rest listed as *Least Concern* by the IUCN. Three species, namely, *Amblyglyphidodon ternatensis*, *Gobiodon axillaris*, and *Chrysiptera hemicyanea*, are *Vulnerable* in the Red List due to declining coral cover globally (IUCN, 2023). Only one highly traded MAT species, *Pterapogon kauderni*, is *Endangered* in the Red List due to its small area of occupancy, severe fragmentation, and continuing decline due to the aquarium trade (Allen & Donaldson, 2007). We note the significant aquaculture production of *Pterapogon kauderni* (Table 2, Rhyne et al., 2017). Both methods showed that damselfishes (Family: Pomacentridae) are least vulnerable among the 306 species assessed. Among the species analyzed in the PSA, the 10 species with the lowest vulnerability scores are classified as ***Least Concern***, except for *Amblyglyphidodon ternatensis*. This species is categorized as *Vulnerable*, primarily due to the global decline in coral cover (IUCN, 2023).

**FIGURE 8:**
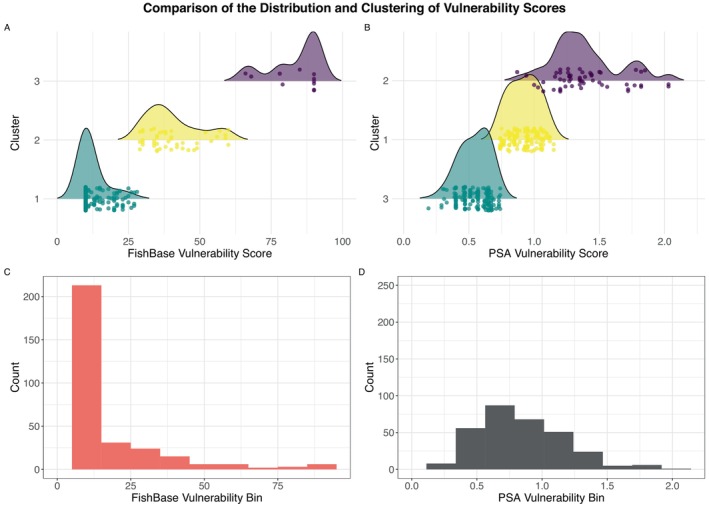
Ridgeline plot of GAM clustering of vulnerability scores from Fishbase (A) and PSA (B). Histogram of vulnerability score distribution (*n* = 10 data bins) from FishBase (C) and the present productivity–susceptibility analysis (PSA) (D) for all species assessed (*n* = 306).

A pair‐wise comparison of the FishBase vulnerability data and this PSA show no strong correlation or linear relationship between the two scores for the 306 fish in the trade assessed (Pearson's correlation = 0.16, *R* ^2^ = 0.022). The FishBase model (Cheung et al., 2005) is much less sensitive to species‐specific characteristics for marine aquarium fish, as 191 species are assessed with a vulnerability of “10,” the lowest possible score in that model. Observing the spread of vulnerability scores between the two models, it is evident the FishBase vulnerability scores are severely left skewed whereas the 202 PSA scores show a normal distribution when breaking the data into 10 equal vulnerability bins (Figure 8). When comparing GMM clustering of vulnerability scores between the two scoring frameworks, there was a very uneven division of species within each cluster skewed by the majority of all datapoints being scored as a 10 or “low vulnerability” in FishBase. The same GMM clustering method can be applied to distinguish scores that are low, medium, and high vulnerability; however, the difference in the distribution of datapoints following a normal curve was only seen for the range of PSA scores, not FishBase (Figure 8). Through clustering, 69.61% of species were categorized as low vulnerability, 12.42% as medium, and only 2.29% as high vulnerability compared to the 18.7% the PSA highlighted as at‐risk species.

Comparing the top 15 most vulnerable species ranked by each model, *Chiloscyllium punctatum*, *Aetobatus narinari*, *Taeniura lymma*, and *Stegostoma tigrinum* are the only overlapping species. Comparing seven key species in the trade allows for a further examination of agreement and divergence between these two models (Table 3). The models severely disagreed on the vulnerability score for four (*Pterapogon kaudernii*, *Paracanthurus hepatus*, *Dascyllus aruanus*, *Pomacanthus imperator*) of these seven well‐studied species. The damselfish, *Dascyllus aruanus*, has opposite vulnerability scores comparing the PSA and FishBase vulnerability scores, respectively (*V* = 0.67, 26) where the main distinction here is the Bangaii Cardinalfish (*Pterapogon kaudernii*) scores lower in FishBase vulnerability than the damselfish, while being in the PSA top 10 highest scoring vulnerability species (*V* = 1.52, 19) (Table 3). There is agreement on *Chromis viridis*, and the aforementioned *Echidna nebulosa*.

**TABLE 3:** Comparison of seven key species vulnerability scores and corresponding vulnerability categories between our PSA and the FishBase vulnerability model. Species are ordered by low to high FishBase vulnerability score.

Species	PSA vulnerability	FishBase vulnerability score	GMM cluster vulnerability	FishBase vulnerability category
*Chromis viridis*	0.67	10	Least	Low
*Pterapogon kauderni*	1.52	19	Most	Low
*Paracanthurus hepatus*	1.35	21	Moderately	Low
*Dascyllus aruanus*	0.67	26	Least	Low to moderate
*Echidna nebulosa*	1.56	60	Most	High
*Pomacanthus imperator*	1.02	68	Moderately	High to very high

## DISCUSSION

The MAT operates both as a fishery and under the greater umbrella of wildlife trade more generally. This distinction poses ethical considerations for fish and fisher welfare, but perhaps more perplexingly presents a data problem. Unlike traditional fisheries which are distinguished by gear type, target species, and geographic location of fishing, the MAT encompasses hundreds to thousands of species and the best available data are collated on the importing side of the supply chain as shipments pass customs and are documented. Due to a lack of catch reporting regulations alongside scant resources for enforcement of any existing fishery management policies in the largest exporting countries, there is very limited data to inform a quantitative assessment of the sustainability of the MAT. There is a notable lack of transparency throughout the MAT supply chain, resulting in a near complete loss of information on harvest location and quantity. Given the severe data limitations on a catch and species level, higher granularity data would be needed to understand if the MAT poses a threat of overharvesting at a national or global scale. In response, we present an updated PSA methodology to systematically evaluate the potential vulnerability of marine aquarium fish species to extinction as a result of harvest practices to supply the MAT.

The latest CITES Conference of Parties sought to understand available monitoring and assessment methods to best address the lingering questions surrounding the current and future sustainability of this fishery. A key outcome of this workshop was the need to develop a robust, rapid assessment to prioritize species within the trade that, due to commercial and biological pressures, may not be suitable for the global aquarium trade under the lens of long‐term sustainability. Here, we refer to the definition of sustainability for the MAT as a “relationship between society and reef that continually sustains and improves the net benefit to the coral reef socio‐ecological system” (Rhyne et al., 2014). The need to develop a risk framework was further motivated by concrete evidence of a well‐researched example of MAT harvest levels driving species to near extinction. The Bangaii Cardinalfish is a highly desirable aquarium fish that happens to be endemic to the Bangaii Archipelago in Indonesia. In the early 2000s, this fish was so popular it was nearly fished to extinction, leading to an IUCN *Endangered* listing and CITES Appendix II listing banning the trade of wild‐caught Bangaii Cardinalfish. To prevent localized depletions or worse, the PSA has been designed to harness existing data to estimate the relative vulnerability of fish species in the MAT and enable local governments and industry leaders alike to take early action to prevent overharvesting of ‘high risk’ species. This is strictly a prioritization framework and does not serve to classify fish into explicit and fixed sustainability categories but can identify species that have a host of characteristics making them resilient or vulnerable to fishing in the MAT.

Our analysis shows the PSA is a robust and accessible tool to predict the vulnerability of fish in the MAT from a holistic and global perspective based on the best available data we have—trade volume. Sustainable species in the MAT can be defined as those with low susceptibility and high productivity, indicating populations can withstand shifts in fishing pressure. To overcome data deficiency, yet limit error propagation, the PSA framework is tailored specifically to the characteristics of MAT fish, especially when considering productivity factors. Additionally, it uses a three‐tiered data‐binning process to buffer trade volume estimates against a large range. In lieu of catch or landings data, we estimated trade volume per species based on the most complete and accurate dataset available (Rhyne et al., 2015) of 2011 imports into the United States. In parallel, the best estimate of population health and probability of resilience relies on understanding the life history traits of marine aquarium fish which are grounded in peer‐reviewed literature. This research builds on existing work (Baillargeon et al., 2020) by expanding the analysis from 32 to 306 species in the trade. Modifications to the framework included introducing dynamic yet data‐rich susceptibility factors which have drastically improved the accuracy of the analysis.

The majority of fishes evaluated by Baillargeon et al. (2020) had low susceptibility and high productivity scores, falling in the “sustainable” category, a product of the most traded species being highly fecund with short lifespans (average *p* = 2.28). Even though we increased our sample size by nearly an order of magnitude, we see similar values in the present analysis (average *p* = 2.46 ± 0.33). The 2020 model showed 93.3% of species evaluated were least vulnerable (HPLS) or moderately vulnerable (MPMS), where this revised model with an expanded dataset declined to 81.4%. Elasmobranchs and eels are the most vulnerable species evaluated, are larger and slower to reproduce than small reef fish while also being targeted as juveniles and having a difficult to expert level aquarium suitability rating. Small, fast reproducing fish such as damselfishes remain the least vulnerable fish in the analysis, and are the most prevalent in the trade by volume. This contrast of life histories within the larger group of species targeted by the aquarium trade has shown that elasmobranchs and eels are in need of greater monitoring and management attention, while only a select few reef fish fall into this category (14%). We suggest that elasmobranchs have their own data‐limited assessment methods developed and applied, as this PSA framework is tailored more specifically toward reef fishes.

Several species vulnerability scores and rank in trade exemplify why this risk assessment is a useful tool to assess current sustainability and prevent future declines due to fishing activity. Although not well represented in trade, *Chrysiptera unimaculata* (*V* = 0.19) has exceptionally low vulnerability and the cumulation of PSA outputs suggests that this species would respond well to increased demand from consumers (rank 190 of 306). *Chiloscyllium punctatum* (*V* = 1.82 where maximum *V* = 2.82) is the highest scoring species on this list that we have reliable trade data for, yet it is not well represented in trade (252 of the 306 species). In this case, due to its low productivity, a consideration of a quota limit may be a next management step to prevent future declines with changes in demand or fishing effort. Conversely, *P. kauderni* is the 13th most vulnerable species evaluated, has the highest vulnerability score exclusive of sharks and eels, and is ranked 9th most imported fish into the United States in 2011. This vulnerability of wild populations led the US federal government to initiate a 4D ruling, unfortunately ignoring the significant numbers of this species being produced in aquaculture in Thailand (Rhyne et al., 2017). The PSA framework enables managers to suggest minimally disruptive management methods to decrease risk based on individual productivity and susceptibility scores to avoid implementing broad, uniform management policies that are not tailored to the fishery.

A limitation shared between FishBase, IUCN, or this assessment frameworks is the limited data available in the literature to support growth‐ and reproduction‐related factors. However, the PSA allows for next common congener estimates, a more thorough search of the literature than FishBase, and finally does not assume all species follow the same growth curves. Another source of uncertainty is the use of trade data (import records) as an indicator of total catch and consumer desirability. Since these data are binned, the uncertainty declines as the exact number of fish does not control model outputs directly. There is a high degree of variability, as shown by the error bars in Figures 3 and 5, between individual species and within a family of species. Species are clustered based on productivity, susceptibility, and vulnerability score which means species that are highly vulnerable due to low productivity fall in the same category as those who have high susceptibility and medium‐high productivity. These error bars are not indicative of uncertainty but rather show the full spectrum of how individual species have the capacity to respond to fishing pressure based on trade dynamics and known life history traits. Lastly, while we acknowledge that many other factors could be influencing the vulnerability of reef fish harvested for the MAT, such as climate change or other anthropogenic stressors, the majority of species traded are not harvested for other fisheries. This PSA has been designed as a precise tool to address the potential threats the MAT specifically places on frequently harvested species.

This improved PSA methodology has two advantages over the IUCN Red List process, making it suitable for determining appropriate management, research, and conservation actions for MAT species. Notably, we were able to calculate a vulnerability score and rank MAT species despite data limitations, including species that were *Data Deficient* in the Red List. Second, this PSA method provides a ranking for a species based on their vulnerability to overfishing in the MAT. Although crucial, the direct impact of the MAT on local coral reef ecosystems is often unavailable (Ochavillo et al., 2004). Within the list of *Least Concern* species in the Red List, further prioritization through the PSA was possible considering the species’ vulnerability in the MAT. When looking at the types of threats considered by each method, the Red List evaluates the probability of extinction of a species with consideration of all potential threats to its global population. In contrast, the PSA provides a vulnerability score associated with a specific threat, which is overfishing due to the global trade. A threatened category in the IUCN Red List does not necessarily equate to high vulnerability to overfishing in the MAT, exemplified by our results. Except for the *Endangered* cardinalfish, *Pterapogon kauderni*, many other MAT species were placed in the *Vulnerable* category due to declining coral cover associated with climate change on the Red List. Our improved PSA methodology and resulting ranking is apt for resource managers, policymakers, and researchers looking for information on species vulnerable to the MAT.

Similarly, the nuance of the PSA assessment can be confirmed when comparing our results to that of FishBase vulnerability outputs (Froese & Pauly, 2023), an automated tool that requires little to no information to be inputted on the user end to obtain the vulnerability score of nearly any fish. The FishBase model is reliant almost entirely on estimated life history parameters rooted in the von Bertalanffy growth equation (Cheung et al., 2005). For a group of diverse, data‐deficient species like those in the MAT, this results in either a homogenization of all fish without taking into account any susceptibility factors, or the fish that do score higher are inflated based on one growth characteristic. Most notably, the Bangaii Cardinalfish (*Pterapogon kaudernii*), the only species demarcated as *Endangered* under the US Endangered Species Act and IUCN Red List, is ranked as low vulnerability (*V* = 19) under the FishBase model. This scoring is directly contradicted by the universal agreement that this fish is endangered due to the wild‐caught aquarium fishery, given its unique life history traits of mouth brooding few young in an endemic range while being in high demand due to their appeal to hobbyists, thus making intensive trade unsustainable for this species. Even the fast growing, highly fecund damselfish *Dascyllus aruanus* (*V* = 26) scores higher than a known endangered species, which is in direct opposition to the known, well‐documented life histories and fishing records of both these fish.

The only consistent alignment between the two frameworks is for data‐rich fish that behave as traditional food fish in terms of both growth pattern and susceptibility to fishing pressure, like the Snowflake moray eel (*Echidna nebulosa*, *V* = 60). To further demonstrate this point, a popular angelfish, *P. imperator*, has consistently fallen into the moderate risk category across multiple PSA frameworks (Baillargeon et al., 2020; Dee et al., 2019; Okemwa et al., 2016). Yet, according to the FishBase model, it scores as highly vulnerable (*V* = 68) across, in line with the dogfish (*Squalus acanthias*) score (*V* = 68), showing a huge discrepancy in scoring for marine aquarium fish under this framework. Key traits of a resilient fish in the marine aquarium fishery: small maximum size, broadcast spawner or high parental investment demersal spawner with high fecundity, wide habitat specificity and large geographic range, are only harvested as juveniles, and are considered easy to care for in a home aquarium. For example, the combination of MAT‐specific productivity factors replaces the reliance on growth metrics derived from length–weight relationships or spawning stock biomass surveys which are commonly drawn upon to assess the status of a fished stock. If a fish does not meet these exact criteria, that does not equate to it being highly vulnerable to overfishing. Instead, the PSA analyzes how well its life history characteristics (productivity factors) are in balance with existing fishing pressure (susceptibility factors). Managers can then interpret PSA results based on the vulnerability score and associated cluster, while identifying if their vulnerability is being driven by productivity or susceptibility factors and can tailor management plans to mitigate risk based on this.

## CONCLUSIONS

We present a robust and accessible risk assessment framework that can be adapted to a diverse range of marine aquarium fish with varied data availability. Given that this data‐limited system operates in some of the most biodiverse coral reefs in the Indo‐Pacific, which are already facing an onslaught of environmental challenges, there is a need to ensure that the marine aquarium industry is providing a positive net benefit to the coral reef socio‐ecological system inclusive of reef and community health. The updates made to the previously published framework for MAT (Baillargeon et al., 2020) ensure that species’ vulnerability calculations avoid overestimation of scores through setting more realistic criteria for scoring, elimination of extremely data‐limited factors, and productivity‐scaling (i.e., volume in trade). We recommend that the 57 fish in the most vulnerable cluster are prioritized for independent fishery assessment at national and regional scales to best understand the threat the MAT poses to their populations, and how to implement management policies to prevent declines.

The PSA is the best‐suited data‐limited assessment tool to identify the level and type of risk that fishing poses to current and future species that are heavily traded. Its flexible yet robust framework incorporates 11 data points, far more than either FishBase or IUCN, into a single suite of vulnerability indicators (productivity, susceptibility, vulnerability score, and cluster) that enable accurate and simple prioritization of species for management. Further, this group of species is of high priority for supplementing wild‐caught with aquacultured fish when possible. The PSA method can be used in data‐limited situations to support IUCN Red List Assessments. The potential impact of this extensive list of vulnerability scores for the top 306 species traded should be viewed as a powerful tool for national and international regulatory bodies, such as CITES or IUCN, to adapt into their risk assessment methodologies when robust datasets are frequently unavailable.

## CONFLICT OF INTEREST STATEMENT

Gabrielle A. Baillargeon is funded by UKRI under a NERC Panorama Doctoral Training Program scholarship at the University of Leeds. CASE partners on this scholarship include: Ornamental Aquatic Trade Association (OATA), Center for Environment, Fisheries, and Aquaculture Science (Cefas), and Mars Inc. The data and statements made herein are exclusively the authors' and do not necessarily reflect that of funding partners. Jemelyn Grace Baldisimo (JGB) collaborated on this study as part of her Virginia Sea Grant Graduate Student Fellowship. JGB is a member of the Philippine Aquatic Red List Marine Ornamentals Committee and IUCN Species Survival Commission. The views expressed in this publication do not necessarily reflect those of IUCN. The designation of geographical entities in this paper, and the presentation of the material, do not imply the expression of any opinion whatsoever on the part of IUCN concerning the legal status of any country, territory, or area, or of its authorities, or concerning the delimitation of its frontiers or boundaries. Michael F. Tlusty advises public aquaria on the sustainability of living collections and co‐developed a tool to assist governments on improving wildlife trade data. Andrew L. Rhyne advises public aquaria on the sustainability of living collections and has formal agreements with aquaria concerning aquaculture production of fish species for display. His laboratory specializes in the production of aquarium fish for the trade. Moreover, he has received funding from pet industry groups and nongovernmental organizations (NGOs) interested in restoration aquaculture, reduction in cyanide fishing, and animal welfare. Rhyne also co‐developed a wildlife trade platform that is currently being tested by a national government as an implementation solution to increase the granularity of wildlife trade data. These relationships could be seen to influence the research presented, although every effort has been made to ensure they have not. The statements made herein are solely the responsibility of the authors.

## Supporting information


Appendix S1.

## Data Availability

Data are available in Zenodo as follows: productivity–susceptibility analysis dataset (Baldisimo et al., 2025, https://doi.org/10.5281/zenodo.17468588); dataset compiled from IUCN data for the accessed species (Baldisimo, 2025, https://doi.org/10.5281/zenodo.17468611); all PSA factor data sources (Wynn et al., 2025, https://doi.org/10.5281/zenodo.17468677). Additionally, volume in trade was extracted from the aquariumtradedata.org database (Rhyne et al., 2015), and these data are compiled in Baillargeon et al. (2026) in Zenodo at http://doi.org/10.5281/zenodo.20035439. Model code (gbaillargeon, 2026) is available in Zenodo at https://doi.org/10.5281/zenodo.18941035.
