# Supplementary material for: Evaluating species at risk in data‐limited fisheries: A productivity–susceptibility analysis for marine aquarium fish
Source: Ecol Appl. 2026 Jun 9;36(4):e70272. doi: 10.1002/eap.70272 (PMC13248878; doi:10.1002/eap.70272)
Supplement: Supplementary file 1 — Appendix S1. [file EAP-36-e70272-s001.pdf]

Appendix S1

Evaluating species at risk in data-limited fisheries: A productivity–susceptibility analysis for marine aquarium fish

Gabrielle A. Baillargeon, Alice A. Wynn, Jemelyn Grace P. Baldisimo, Michael F. Tlusty, Andrew Rhyne

Ecological Applications

Table S1: Scoring matrix for PSA factors for productivity and susceptibility with corresponding weight and scoring bin based on raw data parameters.

**Productivity Factors**	**Score 1**	**Score 2**	**Score 3**	**Factor Weight**
**Maximum Size (cm)[1]**	>60	30-60	<30	2
**Trophic Level[1]**	>3.5	2.5-3.5	<2.5	1
**Breeding Strategy[1]**	Live bearer/mouthbrooder with a fecundity score of 1, or a broadcast/demersal spawner with a fecundity score of 1	Distinct pairing, demersal spawner	Broadcast Spawner, demersal spawner with high parental care, or mouthbrooder with a fecundity score of 2 or 3 (above 1,000)	1
**Fecundity (number of eggs produced yearly)[1]**	<1,000	1,000-15,000	>15,000	2
**Pelagic Larval Duration (days)[1]**	>30	14-30	<14	1
**Susceptibility Factors**	**Score 3**	**Score 2**	**Score 1**	**Factor Weight**
**Volume in trade (scaled on productivity score)[1]**	Greater than 3,000 traded and p=0.77-1.9	Greater than 3,000 traded and p=1.9-2.0<br><br>OR<br>Less than 3,000 and p=0.77-1.9	<3,000 total volume in trade<br>OR<br>p=2-2.3 at any trade volume	2
**Ecological niche + Geographic distribution[2]**	Small geographic range/Narrow habitat specificity	1. Large geographic range/Narrow habitat specificity<br><br>2. Small geographic range/Wide habitat specificity	Large geographic range/Wide habitat specificity	2
**Cyanide Use[1]**	1. Country of Export: Indonesia, Philippines, Vietnam<br><br>2. Family: Chaetodontidae, Pomacanthidae, Acanthuridae	No rating of 2 for this category	Score of 1 if conditions in score 3 do not apply	1
**Maximum Encounterability depth (m)[1]**	<10	10-30	>30	1
**Aquarium Suitability[1]**	"Difficult" care level (grows very large, complex diet, aggressive, high tank mortality rate)	"Moderate" care level	"Easy" care level: (remains at small size, resilient to environmental changes, non-aggressive, less likely to need replacing)	1
**Life cycle stage of harvest<br>1: Recruit, 2: Juvenile, 3: Subadult, 4: Adult**	Harvested at juvenile and adult stages.<br>(1,2,3,4), (1,2,3), (2,3), (2,3,4)	Harvested only at adult stage<br>(3), (3,4), (4)	Only harvested at juvenile stage<br>(1), (1,2), (2)	2

[1]Adapted from Baillargeon, et al (2020), [2]Adapted from (Rabinowitz, 1981)

Table S2: Scoring matrix for scaling of breeding strategy score.

If a species had a breeding strategy score of 1, but was highly fecund with a value >1,000 (i.e. score of 2 or 3), the breeding strategy score was scaled to a 3. Conversely, species with breeding strategy scores of 3, but low fecundity values <1,000 (i.e. score of 1), breeding strategy scores were changed to 1.

Original breeding strategy score	Fecundity value	Scaled breeding strategy score
1	> 1000	3
3	<1000	1

Table S3: Results of primary (3 factor manipulation) and expanded (single weighted and unweighted) sensitivity analysis.

	Vulnerability score decrease when shifting from a score of 1 to 3	Vulnerability score decrease when shifting from a score of 3 to 1
**3 factor manipulation, one factor weighted:**	0.882 (Fecundity, Breeding strategy, PLD)	0.734 (Aquarium suitability, encounterability depth, LCSH)
**Single weighted factor manipulation:**	0.364 (Maximum size)	0.387 (Ecological niche + distribution)
**Single unweighted factor manipulation:**	0.183 (Trophic level)	0.199 (Aquarium suitability)

Table S4: Comparative table of three different clustering algorithms: Gaussian-Mixture Model (GMM) with equal shape, size, and direction, K-means clustering using kmeans R package, GMM VVI with diagonal clustering and varying volume and shape. Both GMM models were run using Mclust package in R. The number of species per cluster (total % of species assessed) is displayed for each model. The average vulnerability score for each cluster centroid across the three models is shown, along with the silhouette coefficient and BIC value for each model. Log likelihood is only compared between the two GMM models.

Number of Species per Cluster			
Cluster Category	**GMM**	**K-Means**	**GMM VVI**
Low Vulnerability	166 (64.3%)	143 (55.4%)	127 (49.2%)
Moderately Vulnerable	85 (32.9%)	83 (32.2%)	93 (36%)
High Vulnerability	7 (2.7%)	32 (12.4%)	38 (14.7%)
Vulnerability of Centroid Center Points			
Low Vulnerability	0.62	0.55	0.54
Moderately Vulnerable	1.07	0.99	0.92
High Vulnerability	1.32	1.02	1.26
Model Validation			
Silhouette Coefficient	0.487	0.457	0.4335
BIC	1208.6	44.64	332.738
Log Likelihood	651.51	-	221.90

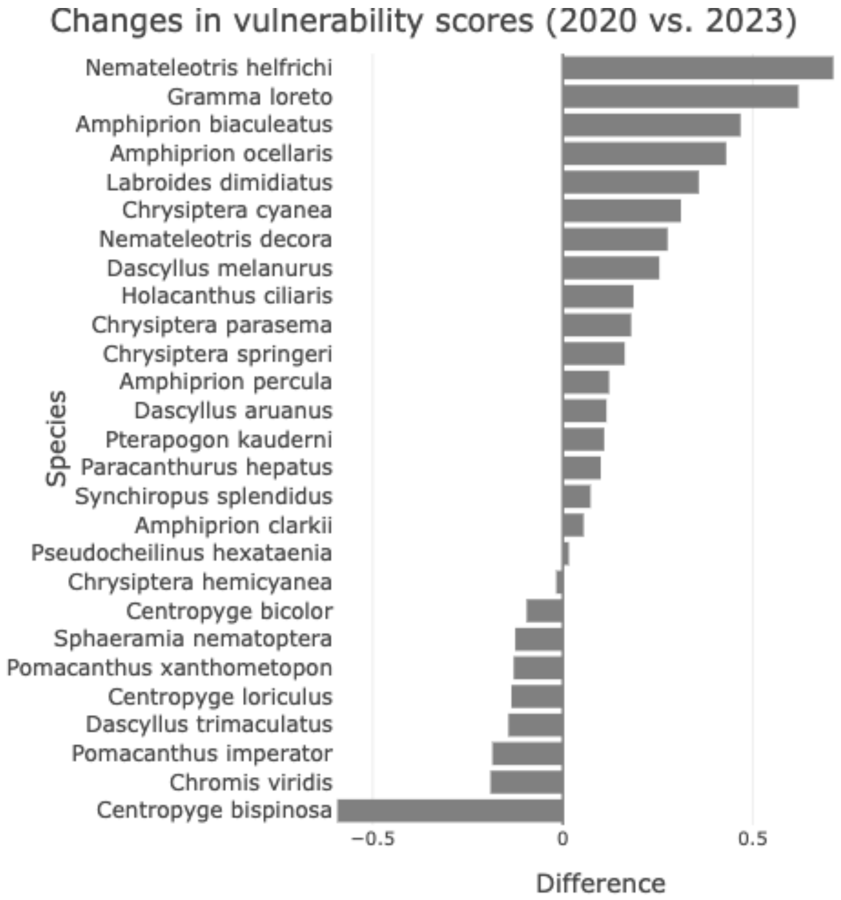

Figure S1: Change in vulnerability scores between Baillargeon et al. (2020), and the present analysis. Species names with asterisks (*) represent fish included only in Baillargeon et al. (2020) due to their position below the 2011 top 258 US imported species and analyzed with our 2024 model.

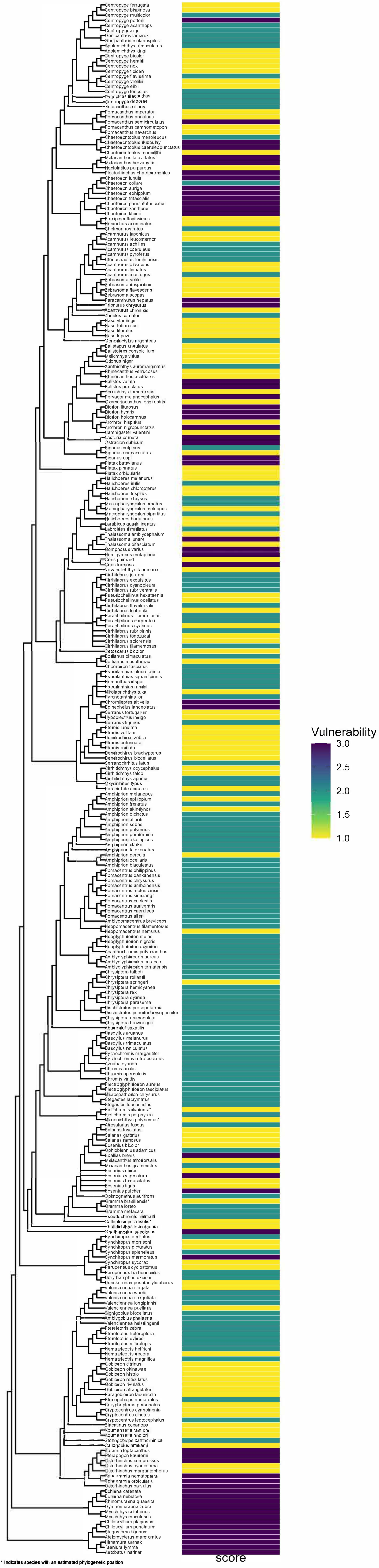

* Indicates species with an estimated phylogenetic position

Figure S2 (previous page): Phylogenetic tree (n=306) of all species assessed using this PSA framework, with a heat map of vulnerability scores layered on the tree based on species vulnerability values. A high-resolution version of this plot is available (Wynn, 2025; https://doi.org/10.5281/zenodo.14833664).

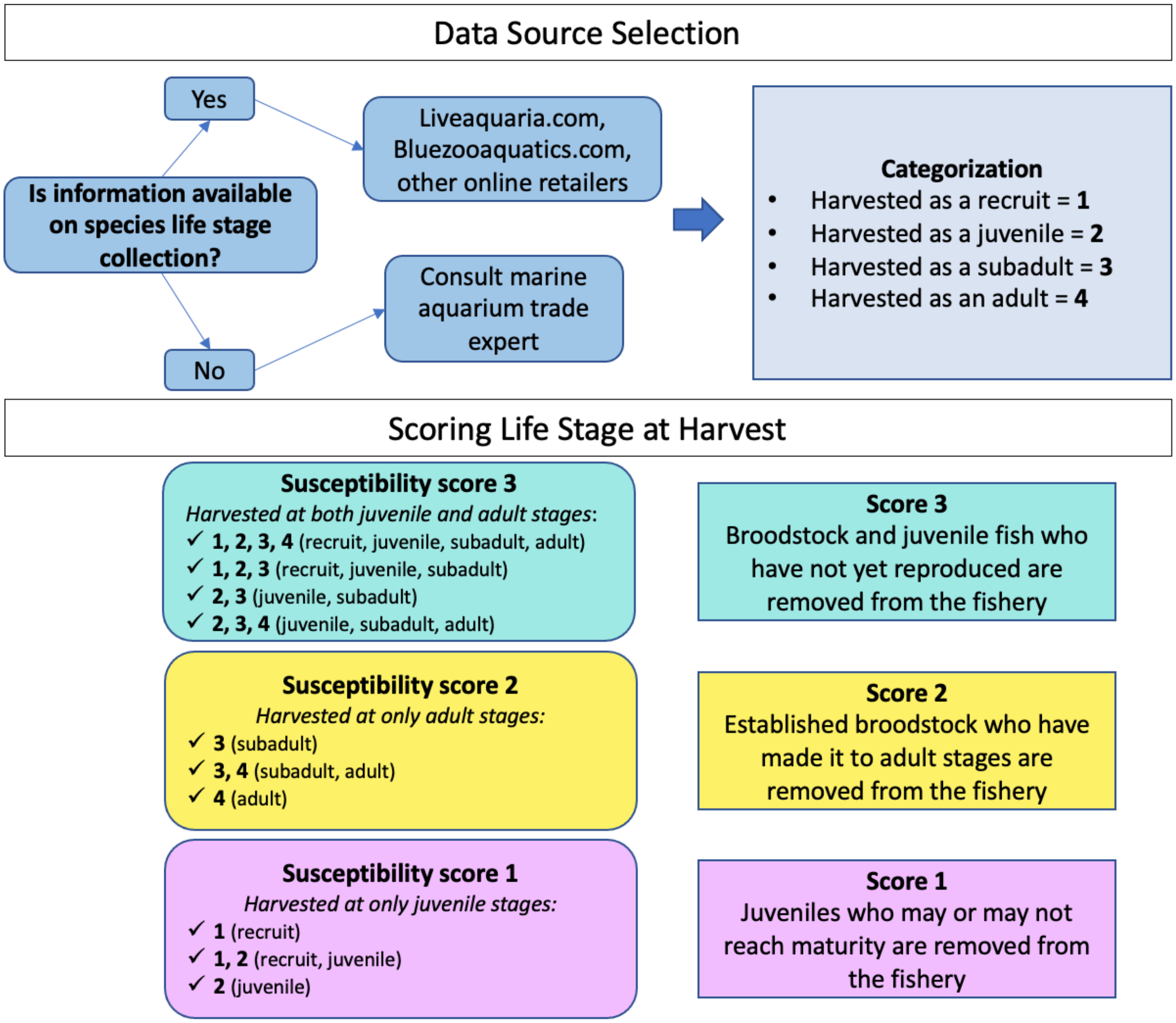

Figure S3: Flow diagram outlining the process of gathering data, assigning a numerical data representation and translating that into the final scoring of the susceptibility factor Life Stage at Harvest.

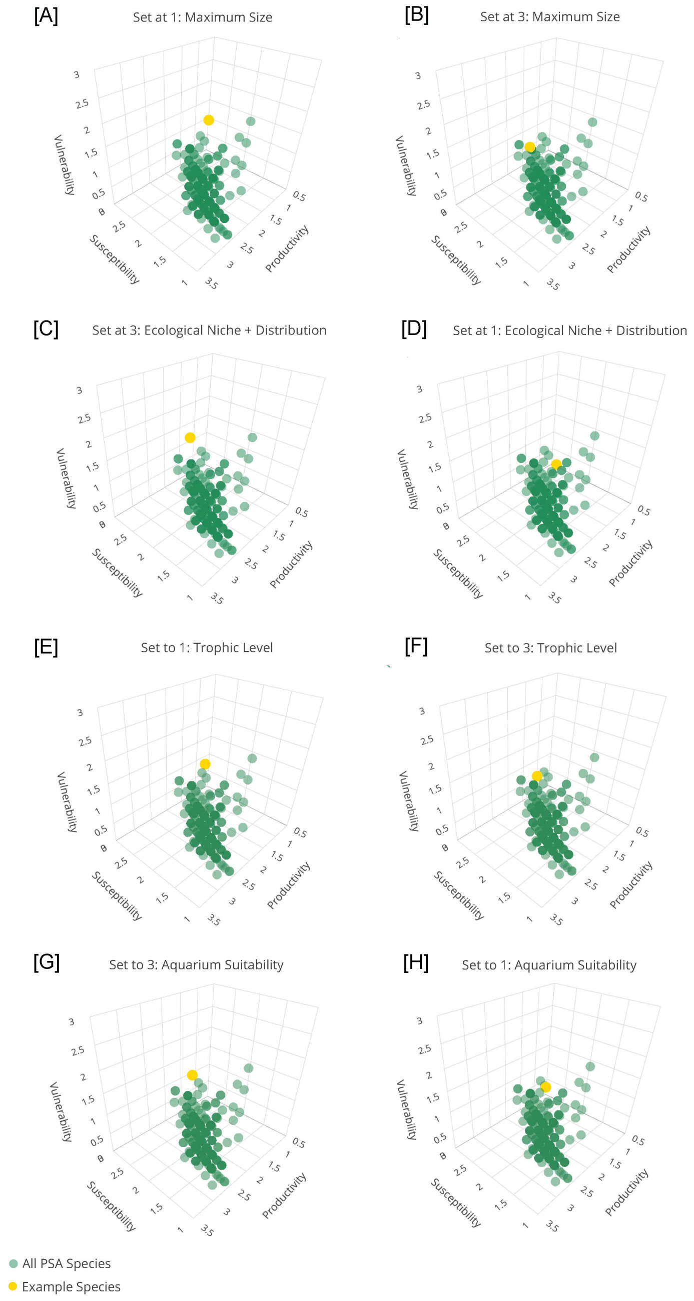

Figure S4: Expanded model sensitivity analysis demonstrating how manipulation of various life history factors influences the position of productivity, susceptibility, and vulnerability for a

hypothetical species. All PSA species are represented by green points, while the hypothetical example species is represented in each plot with a yellow point. Both single weighted and single unweighted factors were tested. For left-hand plots [A, C, E, G] we demonstrate that manipulating factor scores to low productivity, high susceptibility scoring results in increased vulnerability compared to right-hand plots [B, D, F, H] which represent the same factors set to high productivity and low susceptibility scores.

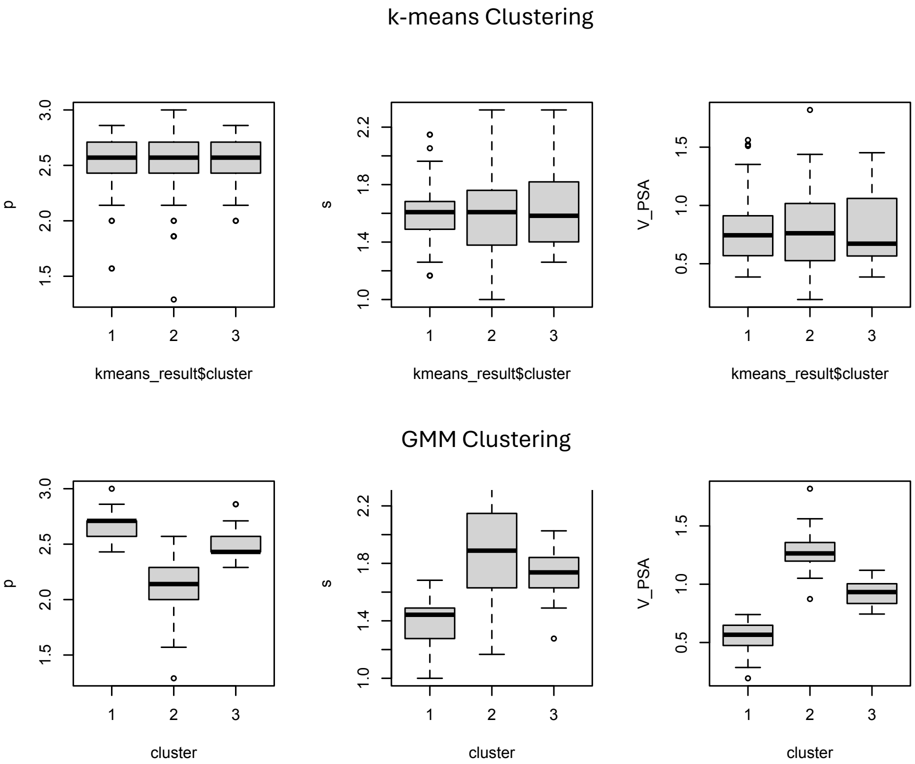

Figure S5. Boxplots showing the distribution of productivity (p), susceptibility (s), and vulnerability (V_PSA) across classification clusters: 1 (least vulnerable), 2 (most vulnerable), and 3 (moderately vulnerable). Comparison of the distribution of scores withing classification clusters between k-means and GMM "VII" McClust clustering algorithms.

References

Baillargeon, G. A., Tlusty, M. F., Dougherty, E. T., & Rhyne, A. L. 2020. Improving the productivity-susceptibility analysis to assess data-limited fisheries. *Marine Ecology Progress Series*, *644*, 143-156.

Rabinowitz D. 1981. Seven forms of rarity. In: Synge H (ed) The biological aspects of rare plant conservation. Wiley, New York

Wynn, A. 2025. Phylogenetic Tree for 306 Species Vulnerability. Zenodo. https://doi.org/10.5281/zenodo.14833664
